# *Shewanella* sp. T2.3D-1.1 a Novel Microorganism Sustaining the Iron Cycle in the Deep Subsurface of the Iberian Pyrite Belt

**DOI:** 10.3390/microorganisms10081585

**Published:** 2022-08-06

**Authors:** Guillermo Mateos, Adrián Martínez Bonilla, Sofía de Francisco de Polanco, José M. Martínez, Cristina Escudero, Nuria Rodríguez, Irene Sánchez-Andrea, Ricardo Amils

**Affiliations:** 1 Centro de Biología Molecular Severo Ochoa, Nicolás Cabrera 1, 28049 Madrid, Spain; 2 Centro de Investigaciones Biológicas, Ramiro de Maeztu 9, 28040 Madrid, Spain; 3 Centro de Astrobiología (CAB-INTA), 28850 Torrejón de Ardoz, Spain; 4 Laboratory of Microbiology, Wageningen University & Research, Stippeneng, 46708 Wageningen, The Netherlands

**Keywords:** *Shewanella putrefaciens*, deep subsurface, hydrogen, iron, NDFO

## Abstract

The Iberian Pyrite Belt (IPB) is one of the largest deposits of sulphidic minerals on Earth. Río Tinto raises from its core, presenting low a pH and high metal concentration. Several drilling cores were extracted from the IPB’s subsurface, and strain T2.3D-1.1 was isolated from a core at 121.8 m depth. We aimed to characterize this subterranean microorganism, revealing its phylogenomic affiliation (Average Nucleotide Identity, digital DNA-DNA Hybridization) and inferring its physiology through genome annotation, backed with physiological experiments to explore its relationship with the Fe biogeochemical cycle. Results determined that the isolate belongs to the *Shewanella putrefaciens* (with ANI 99.25 with *S. putrefaciens* CN-32). Its genome harbours the necessary genes, including *omc*A *mtr*CAB, to perform the Extracellular Electron Transfer (EET) and reduce acceptors such as Fe^3+^, *nap*AB to reduce NO_3_^−^ to NO_2_^−^, *hyd*AB to produce H_2_ and genes *sir*A, *phs*ABC and *ttr*ABC to reduce SO_3_^2−^, S_2_O_3_^2−^ and S_4_O_6_^2−^, respectively. A full CRISPR-Cas 1F type system was found as well. *S. putrefaciens* T2.3D-1.1 can reduce Fe^3+^ and promote the oxidation of Fe^2+^ in the presence of NO_3_^−^ under anaerobic conditions. Production of H_2_ has been observed under anaerobic conditions with lactate or pyruvate as the electron donor and fumarate as the electron acceptor. Besides Fe^3+^ and NO_3_^−^, the isolate also grows with Dimethyl Sulfoxide and Trimethyl N-oxide, S_4_O_6_^2−^ and S_2_O_3_^2−^ as electron acceptors. It tolerates different concentrations of heavy metals such as 7.5 mM of Pb, 5 mM of Cr and Cu and 1 mM of Cd, Co, Ni and Zn. This array of traits suggests that *S. putrefaciens* T2.3D-1.1 could have an important role within the Iberian Pyrite Belt subsurface participating in the iron cycle, through the dissolution of iron minerals and therefore contributing to generate the extreme conditions detected in the Río Tinto basin.

## 1. Introduction

The study of extreme environments has expanded the established boundaries for life and created many new questions of astrobiological interests such as how life could develop in other planets such as Mars [1]. Río Tinto is considered a Martian analogue due to its mineralogy and geochemistry [2]. This lentic ecosystem draws out from the Iberian Pyrite Belt (IPB), as one of the biggest reservoirs for Fe and S, mainly in the form of Pyrite (FeS_2_), and it has been a mining hotspot for over 5000 years. Its waters show pH values below 3 and high metal concentrations [3] that have been traditionally attributed to the exploitation of the mineralogical resources in the IPB. 

The study of the deep subsurface has been traditionally tied to the geological search for minerals of economic interest. However, the recent discovery of microorganisms associated to hard rocks has increased the interest in the exploration of the continental subsurface and subseafloor. These studies range from the evaluation of its diversity [4,5], the study of microbial community dynamics [6] or the interaction with the environment [7,8]. An exciting feature is its isolation from light and therefore from the photosynthetic-based carbon cycle of the surface, hence this microbial communities are known as the dark biosphere [9,10,11]. The study of the deep subsurface is not an easy endeavour, especially due to logistical reasons. Nowadays, biological studies of the subsurface aim for a combination of techniques using different approaches to produce more resilient results. The Iberian Pyrite Belt Subsurface Life Detection (IPBSL) project aimed to prove that the high concentration of ferric iron in the Río Tinto basin is the result of an underground bioreactor operating in the subsurface of the IPB. Deep subsurface samples were drilled up to 630 m, and complete physicochemical and mineralogical profiles of the boreholes were generated [12,13]. Several microbial enrichments with relevant metabolisms were obtained and from there a number of relevant microbes have been isolated, including the strain T2.3D-1.1 from a 121.8 m depth core from an anaerobic denitrifying culture. Its genome was sequenced revealing that it belongs to the *Shewanella* genus [14]. Members of the *Shewanella* genus are Gram-negative bacteria, widely spread in nature, with most of its representatives known as heterotrophs, facultative anaerobes and fortuitous fermenters [15]. Nevertheless, the main trait that has sparked interest around this genus is the Extracellular Electron Transfer (EET), which allows the reduction of a wide variety of external electron acceptors such as Fe, Mn, Tc and V minerals or even U [16]. So far, there are three known ways through which the EET could take place in the *Shewanella* members, (1) direct contact between the cell and the external acceptor, (2) the production of soluble electron shuttles such as flavins or (3) the production of outer membrane protrusions known as nanowires *sensu lato* [17].

We aimed to gain knowledge on the metabolic potential of strain T2.3D-1.1, at the same time looking for hypothetical ecological niches and roles within biogeochemical cycles. State of the art genome analysis tools such as *variant calling analysis* were used to generate in silico predictions followed by physiological experiments to confirm them. Additionally, physiological experiments were also performed to evaluate its ability to modify the redox state of Fe, as a key element in the IPB and the Río Tinto ecosystem [18]. 

## 2. Materials and Methods

### 2.1. Shewanella sp. T2.3D-1.1 Isolation Context

Isolation conditions and methodology for the IPBSL project and for its different isolates can be found here [12,14,19]. Specific isolation conditions and location for T2.3D-1.1 strain isolation can be found here as well [14].

### 2.2. Shewanellla sp. T2.3D-1.1 Genome Annotation 

*Shewanella*’s sp. T2.3D-1.1 reads, assembly and genome annotation are available in the GenBank database with the accession number GCA_902728295.3. Further information on the genome assembly process can be found elsewhere [14]. CRISPR-Cas clusters were studied with the CRISPRCasMeta tool [20]. CRISPR sequences without associated Cas and without a minimal distance group of 5000 were discarded when examining the results. Full display of results can be visualized in the CRISPRCasFinder Viewer tool using Supplementary Material S1. Non-annotated genes were further examined with the BLAST suite from the NCBI server [21]. MetaCyc was also used in order to visualize the genomic data and to map genes to model microorganisms [22].

### 2.3. Shewanella spp. Genome Accessions Numbers

From the GenBank database, a total of 73 genomes were downloaded. This includes 16 non-type strains *Shewanella putrefaciens* genomes and 57 *Shewanella* type strains. A complete list including the accession numbers is available in Supplementary Table S1. 

### 2.4. Phylogenomic Analysis and Taxonomic Classification

Digital DNA-DNA Hybridization (dDDH) calculations were made through the DMSZ GGDC (Genome to Genome Distance Calculator) web page using default parameters. Average Nucleotide Identity (ANI) was calculated using the FastANIv1.32 package from the Anaconda repository [23] with default parameters. Pangenome analysis were made using the Roary tool available on the Galaxy|Eu server with default parameters [24]. The phandango web page was used to visualize results obtained from Roary and to add metadata [25]. Strains and metadata employed on the construction of the pangenome are available in Supplementary Table S2. Consensus phylogenomic tree with 92 core genes was made with the UBCGv3.0 pipeline using all the *Shewanella*’s genomes used in dDDH, ANI and Roary analyses [26].

### 2.5. Variant Calling Analysis 

*Shewanella* sp. T2.3D-1.1’s reads were aligned against the genome of *Shewanella putrefaciens* CN-32 using Bowtie2 v2.3.5.1 with default options for the “build” and “x” commands. Pre-processing was carried out with Picard v2.22.3 using “MarkDuplicates” with the “OUTPUT_DUPLICATE_PIXEL_DISTANCE” set to 250 and “AddOrReplaceReadGroups” with default options before creating an index with “BuildBamIndex”. Variants were determined using GATK v4.1.6.0 with the “HaplotypeCaller” command changing the “ploidy” option to 1, and filtered with VcfFilter within Biopet scaffold v0.9.0. Parameters included “minSampleDepth”, “minTotalDepth” and “minGenomeQuality” set to 9, 9 and 10, respectively. Resulting variants were annotated with SnpEff v4.3 [27] and can be checked in Supplementary Tables S3 and S4. Annotated variants have been sorted according to the metabolic pathways on KEGG using ClusterProfiler v3.1.4.3 [28] with KEGG.db database [29], in RStudio v1.2.5033 (Supplementary Table S5). In addition, the study of the pathway distribution was checked with the KEGG mapper web service [30]. 

### 2.6. BtuB Protein Analysis 

The TonB dependent corrinoid transporter, BtuB¸ is involved in the uptake of different forms of vitamin B_12_ in Gram negative bacteria [31] such as *Shewanella*. The study of the BtuB protein was carried out using BtuB of *Escherichia coli* (CAD6020855.1) and *Shigella dysenteriae* (RIH46231.1) as references. The MEME suite was used for discovering novel and ungapped motifs [32]. We applied the discriminative mode with *E. coli* and *S. dysenteriae* as control sequences and *Shewanella*’s BtuB proteins as primary. The number of expected motifs was set to 10. BlastP suite from the NCBI webpage was used to study and compare the domains found in the proteins. Clustal Omega from the EMBL webpage [33] was used with standard parameters to align all the *Shewanella* BtuB proteins found on the Genbank database and BtuB proteins from *E. coli* and *S. dysenteriae*. The output was represented as a phylogenetic tree and edited with iTOL. Amino acid sequences are included in Supplementary Material S2. 

### 2.7. Metal Tolerance

The metal tolerance assay of *Shewanella* sp. T2.3D-1.1 was carried out in TSA agar medium supplemented with different concentrations of metal salts. The salts used were CuSO_4_, CoSO_4_, CrCl_3_, ZnSO_4_, CdSO_4_, Pb(NO_3_)_2_ and NiCl_2_ and the concentrations account for 0.1 mM, 0.5 mM, 1 mM, 5 mM, 7.5 mM and 10 mM. Metal salts were added to the TSA medium after autoclaving and before gelation from filter-sterilized 0.5 M stocks. Each experiment was performed by triplicates plus a negative control. The plates were incubated at 25 °C. Plates were examined daily for 4 weeks.

### 2.8. Anaerobic Culture and Electron Acceptors

*Shewanella* sp. T2.3D-1.1 was grown anaerobically in a modified version of the Shewanella Basal Medium (SBM) [34]. No mineral mix was added, and the vitamin mix used is as described by [35]. PIPES [2,2′-(Piperazine-1,4-diyl) di(ethane-1-sulfonic acid)] was used as buffer for all anaerobic cultures with at 5 mM. Lactate (16 mM) was added as the electron donor and NO_3_^−^ (10 mM), TMAO (20 mM), DMSO (25 mM), S_2_O_3_^−^ (10 mM) or S_4_O_6_^2−^ (10 mM) as electron acceptors. Media were prepared anaerobically by boiling distilled water and flushing with N_2_. Serum bottles were sealed with butyl-rubber stoppers and crimped with an aluminium cap. The pH of the media was adjusted to 7 after autoclaving with an anaerobic stock of NaHCO_3_ 1.0 M. Growth was confirmed by looking at the cultures with an optical microscope with the 100× objective lens.

### 2.9. Iron Reduction and Oxidation Assays

For the iron reduction assay, *Shewanella* sp. T2.3D-1.1 was grown in anaerobic conditions in a different modified SBM that contained 0.14 g/L of KH_2_PO_4_ as the only phosphate source, in order to avoid precipitations. Lactate (16 mM) was added as the electron donor and Fe^3+^-citrate (4 mM) was added as the electron acceptor. Concentrations were kept to a 4:1 ratio of lactate to Fe^3+^-citrate as reported by [36] and pH was adjusted to 7 after autoclaving using an anaerobic stock of NaHCO_3_ 1 M. The oxidation assay was carried out in a modified medium PaFe2N2 [37] supplemented with 0.46 g/L of NaCl, 10 mM of lactate, 5 mM of NaNO_3_ and with anaerobic FeCl_2_ from a 0.5 M filter-sterilized solution to a final concentration of 4 mM. pH adjustment was carried out with an anaerobic stock of NaOH 0.5 M. Then, the medium was let to settle for 48 h allowing the precipitation of vivianite ((Fe^2+^)_3_(PO_4_)_2_·8H_2_O) and siderite (FeCO_3_) [38]. Finally, the medium was transferred to anaerobic serum bottles using anaerobic and sterile syringes and filtrating it with 0.22 μm pore-size filters to remove the aforementioned precipitates. 

The assays were carried out in triplicates with 6.25% of inoculum *Shewanella* sp. T2.3D-1.1from an anaerobic culture grown in SBM or PaFe2N2 without FeCl_2_. Negative controls were inoculated with the same volume of sterile medium (SBM or PaFe2N2 without iron). The bottles were incubated at 25 °C without shaking. Samples were withdrawn every 24 h during the first week and every 48 h during the second week from the serum bottles using syringes. Then, the ferric and ferrous iron concentrations were quantified using a modified version of the dipyridyl method [39,40]. Briefly, 10 μL of sample are mixed with 40 μL of hydroxylamine as a reductant for Fe^3+^ to obtain a measure of the total iron of the sample (10% in HCl 1 M) or with 40 μL of water to measure the Fe^2+^. Then 100 μL of sulfamic acid (40 mM in HCl 1 M) are added to acidify the pH and remove NO_2_^-^ from the solution [41]. After 30 min, 150 μL of ammonium acetate (28%) and 200 μL of 2,2′-dipyridyl (0.5% in absolute ethanol) are added and incubated for 5 min. Finally, 1.5 mL of miliQ water are added to reach a final volume of 2 mL. Then, spectrometric measurements were obtained with a ThermoFisher Evolution 300 UV-vis spectrophotometer at 520 nm and the total iron concentration was determined using a calibration curve. For the ferrous iron quantification, 40 μL of miliQ water is added instead of hydroxylamine. The ferric iron concentration is the difference between the total iron and the ferrous iron concentrations. For the Fe^3+^ reduction experiments, Fe concentration was measured every 24 h for 7 days.

### 2.10. H_2_ Production and Measurements

To test if H_2_ could be produced by *Shewanella* sp. T2.3D-1.1, lactate (20 mM) or pyruvate (20 mM) were used as electron donors and Fumarate (10 mM) as the electron acceptor [42]. Media were prepared following the same protocol from the last section. To inoculate the media, 39 mL serum bottles were filled with 20 mL of the medium using a sterile and anoxic syringe that had been previously flushed with N_2_. The bottles were incubated at 25 °C without shaking and sealed with PTFE stoppers with valves for chromatographic analysis. Gases from the headspace were analyzed with a Bruker Series Bypass 450 GC chromatograph. The chromatograph was equipped with a column CP2056 0.6 m × 1/8” Ultimetal Cromsorb GHP 100–120 mesh, a column CP81073 0.5 m × 1/8” Ultimetal Hayesep Q 80–100 mesh, and a detector TCD at 200 °C for the detection of H_2_.

## 3. Results and Discussion

### 3.1. Genomic Characterization

#### 3.1.1. Genotypic Analysis 

The *Shewanella*’s sp. T2.3D-1.1 genome is comprised of a circular chromosome of 4.68 Mb and a putative plasmid of 23.8 Kb, both of which were inspected to further elucidate its role within the subsurface of the IPB. The chromosome shows a GC content of 44.42%, a total of 4195 features of which 4068 are coding DNA sequences. Only 2327 have been annotated as proteins while 1741 are regarded as hypothetical proteins. There are 108 tRNA genes, 10 rRNA genes and three repeat regions that are associated with CRISPRs [14]. The GC content remains within expected values for the *Shewanella* genus which range from 38% to 54% and for the *S. putrefaciens* species which range between 43% and 49% [15].

**Figure 1:**
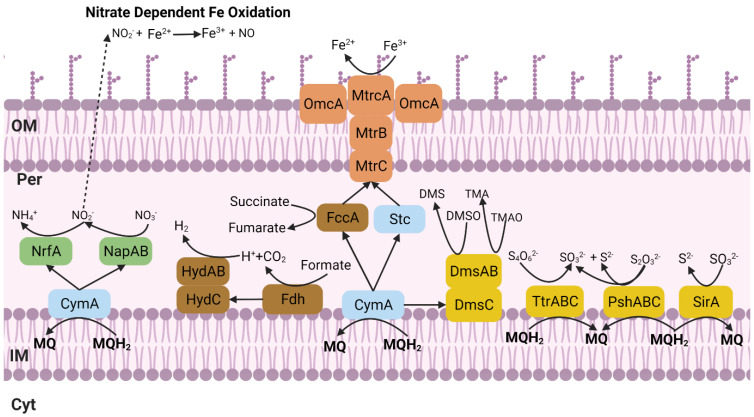
Schematic representation of the membrane of *Shewanella* sp. T2.3D-1.1 and enzymes involved in the usage of different electron acceptors. Solid black arrows represent electron transfer reactions. Colored text boxes are associated to different biogeochemical cycles. Green is assigned to the nitrogen cycle, orange to the iron cycle, yellow to the sulphur cycle and brown to the carbon cycle; blue is for electron transfer proteins. Created with Biorender.

The survival strategies of microorganisms thriving in the subsurface remain poorly understood. The subsurface is still clouded by mystery regarding how microorganisms survive. We have extensively sought in *Shewanella* sp. T2.3D-1.1’s chromosome for genes that would allow its development in these environments. Supplementary Table S6 contains all the annotated genes that are mentioned and discussed in this section. When focusing on its possible roles within the nitrogen biogeochemical cycle, genes found that are related to this biogeochemical cycle would allow this isolate to reduce NO_3_^−^ to NO_2_^−^ and NO_2_^−^ to NH_4_^+^ thus *Shewanella* sp. T2.3D-1.1 could perform NO_3_^−^ dissimilatory reduction to NH_4_^+^ (Figure 1). The genome includes four copies of the periplasmic NO_3_^−^ reductase *nap*A (SHEWT2_00327, SHEWT2_01790, SHEWT2_02339, SHEWT2_03602), one copy of the electron transfer subunit gene *nap*B (SHEWT2_02342) and one copy of the *nap*C (SHEWT2_01792) gene that codes a regulator protein for the electron flow from quinones to the NapAB complex. This also includes two copies of the *nrf*A (SHEWT2_01079, SHEWT2_02986) gene that codes the catalytic subunit of the NO_2_^−^ reductase and one copy of *nrf*B (SHEWT2_03578) that mediates electron transfer to *nrf*A when translated. Although *nrf*H was not annotated, two copies of *nrf*A were identified, where one of them could possibly act as the missing *nrf*H. *Shewanella oneidensis* MR-1 lacks a *nrf*H gene, yet it is capable of reducing NO_2_^−^ to NH_4_^+^ with just one copy of the *nrfA* gene [43]. In the case of the genus *Shewanella*, there is another tetraheme cytochrome, known as *cym*A (SHEWT2_04119), that can also transfer electrons to NrfA [44]. The *cym*A gene is also involved as an intermediary protein in the reduction of electron acceptors such as fumarate, urocanate, As^5+^ or in mechanisms such as the EET [17]. There is also *hcp* (SHEWT2_01360), a gene that encodes a hydroxylamine (NH_2_OH) reductase. Biological production of NH_2_OH in anoxic environments can occur as a short-lived intermediary in the anammox metabolism [45]. Since *Shewanella* sp. T2.3D-1.1 lacks the necessary genes to perform the nitrification or anammox, these substrates could come from anammox bacteria from the IPB’s subsurface (Escudero personal communication).

Denitrification in the *Shewanella* genus is not a common trait, few strains, including *S. amazonensis* SB2B, S. *denitificans* OST5127 and *S. lohiica* PV4, have been reported to reduce NO_3_^−^ to N_2_ [46,47,48]. These isolates employ either the *nar* or the *nap* cluster to reduce NO_3_^−^ to NO_2_^−^, the *nir* cluster to reduce NO_2_^−^ to NO, the *nor* cluster to reduce NO to N_2_O and the *nos* cluster to reduce N_2_O to N_2_. Although *S. amazonenesis* is capable of denitrification without the presence of the *nos* cluster [46,49]. In the case of the strain T2.3D-1.1 *nar* and *nir* clusters have not been found and the *nor* and *nos* clusters are incomplete. This suggests that *Shewanella* sp. T2.3D-1.1 is not able to catalyze the reduction from NO to N_2_. Curiously, genes such as *nos*DFYL (SHEWT2_04147, SHEWT2_04148, SHEWT2_04146 and SHEWT2_04149), that encode a putative ABC transport system for Cu that aids the maturation of the N_2_O reductase, are present although the *nos*Z coding gene for the N_2_O reductase has not been found [50,51]. On the other hand, genes involved in the reduction of NO have been found in the chromosome, with four copies of the NO reductase transcriptional regulator *nor*R (SHEWT2_00349, SHEWT2_02198, SHEWT2_02666 and SHEWT2_03093) and one copy of another transcriptional regulator known as *nor*G (SHEWT2_02621). Although the NO reductase genes (*nor*BC) were not annotated, when using the amino acid sequence of NorB (WP_006081607.1) from *Shewanella baltica* as the query, a Cbb3-type cytochrome c oxidase subunit CcoN1 from *Shewanella* sp. T2.3D-1.1 (SHEWT2_01844) showed a 97.28% of percentage identity with the query. Although it cannot be concluded that this isolate could denitrify generating N_2_, it remains to be confirmed if an alternative route could be carried out by *Shewanella* sp. T2.3D-1.1. Representatives of the genus, including *S. oneidensis* MR-1, can enhance denitrification in co-culture with *Paracoccus denitrificans* through the production of nanotubes between both microorganisms [52]. This *Shewanella* strain shows a similar genetic composition when it comes to the nitrogen cycle, being unable to reduce NO or N_2_O. *Shewanella* sp. T2.3D-1.1 could play an equivalent role with denitrifying microorganisms of the IPB’s subsurface, including the *Rhizobium* sp. strain T2.30D-1.1 [53], *Rhodoplanes* sp. strain T2.26MG-98 [54] or *Pseudomonas* sp. T2.31D-1.1 [55] all of them isolated from the IPB’s subsurface and which have the full denitrification gene array annotated in their genomes. 

The IPB is one of the biggest metal sulphide reservoirs on Earth [56], thus the sulphur cycle must play a crucial role in its geomicrobiology with great potential for biotechnological applications. Several genes with a relevant role in this cycle were detected in *Shewanella* sp. T2.3D-1.1’s (Supplementary Table S6). The *cys* operon (SHEWT2_01034, SHEWT2_02696, SHEWT2_02782, SHEWT2_00316, SHEWT2_00338, SHEWT2_00339, SHEWT2_00340, SHEWT2_00318, SHEWT2_00319, SHEWT2_02699, SHEWT2_00873, SHEWT2_03045, SHEWT2_01032, SHEWT2_02698, SHEWT2_01033, SHEWT2_02697 and SHEWT2_01894), responsible for sulphate (SO_4_^2−^) assimilation and transport is encoded in the genome suggesting that *Shewanella* sp. T2.3D-1.1 can use SO_4_^2−^ as a sulphur source for anabolism and not just sulphide (S^2−^) or reduced sulphur sources. Dimethyl Sulfoxide (DMSO) could also be reduced to Dimethyl Sulphide (DMS) as the genes *dms*A (SHEWT2_01137) and *dms*B (SHEWT2_01138) are present in the chromosome (Figure 1). None of the genes responsible for the dissimilatory reduction of SO_4_^2−^ have been found in the chromosome. Nonetheless, the chromosome of *Shewanella* sp. T2.3D-1.1 holds several genes that can lead to H_2_S production. The *sir*A (SHEWT2_02032, SHEWT2_04142) gene codes an enzyme that catalyzes the reduction of sulphite (SO_3_^2−^) to H_2_S, and in the case of *S. oneidensis* MR-1, it has been reported that this enzyme would act in a dissimilatory fashion as a new type of SO_3_^2−^ reductase [57]. *S. oneidensis* MR-1 can also reduce thiosulphate (S_2_O_3_^2−^) and elemental sulphur (S^0^) with the PsrBAC proteins, a homologue of the PhsABC complex, yielding H_2_S and SO_3_^2−^ [58]. Genes coding the *phs*ABC (SHEWT2_01154, SHEWT2_01153 and SHEWT2_01152) cluster have been found in the chromosome of *Shewanella* sp. T2.3D-1.1 as well. The TtrABC proteins catalyzes the reduction of tetrathionate (S_4_O_6_^2−^) to S_2_O_3_^2−^ which were also annotated in the chromosome (SHEWT2_01103, SHEWT2_01101 and SHEWT2_01102) [59]. Although this strain seems incapable of reducing SO_4_^2−^ to H_2_S, it can potentially use many other forms of S as electron acceptors that most likely will end up as H_2_S (Figure 1).

Regarding the carbon cycle, *Shewanella* sp. T2.3D-1.1 has genes that would allow it to perform the mixed-acid fermentation. Therefore, this strain could hypothetically grow fermenting pyruvate producing succinate, 2-oxoglutarate, acetate, ethanol and/or lactate. Past studies of the IPB have shown that there are relatively high concentrations of organic acids such as acetate, formate and oxalate [60] which could be a product of metabolisms such as pyruvate fermentation and could be used as an energy and carbon source to thrive in subsurface. *S. oneidensis* MR-1 lacks the coding genes for the canonical formate hydrogen lyase complex involved in the production of H_2_ and CO_2_ but can produce H_2_ through alternative hydrogenases. This pathway depends on the *hyd*AB and *fdh* genes that code a periplasmic hydrogenase that would produce H_2_ [42]. Similar genes have been found in the genome of our strain including the *hyd*AB (SHEWT2_03151 and SHEWT2_03152) and several *fdh* genes (SHEWT2_03597, SHEWT2_03601, SHEWT2_03598 and SHEWT2_03609). Nevertheless, genes involved in the maturation of the protein such as *hyd*EFG have not been identified. Experiments to proof the production of H_2_ are described on the Phenotypic characterization section (Figure 1). 

The EET pathway has been thoroughly studied in the *Shewanella* genus, and the corresponding genes were found in *Shewanella* sp. T2.3D-1.1 as well. Electrons can be transferred through the production of extracellular flavins, direct contact between the cell and the extracellular electron acceptor and through distant contact between the nanowires and the external acceptor [17]. Identified genes that were found include *cym*A (SHEWT2_04119), *cct*A (SHEWT2_01539 and SHEWT2_03879) and *fcc*A (SHEWT2_01959 and SHEWT2_02057) which are responsible for the transport of electrons along the periplasm to the Metal-Reducing (MTR) complex, encoded by *mtr*CAB (SHEWT2_00771, SHEWT2_00773 and SHEWT2_01140) and *omc*A(SHEWT2_00770, SHEWT2_01347 and SHEWT2_00866) that also have been found in the chromosome [16]. Additionally, a copy of the *yee*O (SHEWT2_02378) gene has been identified in the chromosome. The protein YeeO is a predicted flavin exporter located in the inner membrane of the cell [61] and to the best of our knowledge, has not been described in *Shewanella* so far. For flavins to be exported, first its synthesis must occur intracellularly. The pathway involves the synthesis of riboflavin, FMN and/or FAD from GTP and ribulose 5′-phosphate. This includes the *rib* (SHEWT2_00417, SHEWT2_00086, SHEWT2_03811, SHEWT2_00083, SHEWT2_01869, SHEWT2_01486, SHEWT2_03014, SHEWT2_00083, SHEWT2_00085 and SHEWT2_00084) cluster of genes and the *fre* (SHEWT2_04023) gene which can be found in *Shewanella* sp. T2.3D-1.1’s chromosome (Supplementary Table S6). Thus, harnessing these genes *Shewanella* sp. T2.3D-1.1 should be able to perform the EET in all the known mechanisms. The EET would allow to access distant external acceptors since nanowires have been measured to be up to 100 μm [62], but also dissolve minerals [63] which could ease other microorganisms to use the dissolution products. Therefore, chemolithotrophy would take the place of photoautotrophy as primary production in the deep subsurface aided by the action of bacteria that can solubilize minerals making their products biologically available.

Even though viruses are known to be crucial for population and ecosystem dynamics, there is little evidence about their role in the deep subsurface. Daly et al. (2019) showed that viruses from several 2.5 km deep hydraulic fractured shales regulate and interact with the predominant populations of bacteria [64]. Microorganisms living in the subsurface show genes and systems related to the defence against these biological entities such as the CRISPR-Cas system [64,65]. This is also the case with *Shewanella* sp. T2.3D-1.1, which has two CRISPR-Cas clusters (Supplementary Material S1). The first one has a CRISPR-Cas system 1-F type since its genome includes genes *cas*1 (SHEWT2_00722), *cas*3 (SHEWT2_00723), *cas*6f (SHEWT2_00727), *csy*1 (SHEWT2_00724), *csy*2 (SHEWT2_00725) and *csy*3 (SHEWT2_00726) [66] and 2 CRISPR cluster one with 1 spacer and the second one with 41 different spacers. The other CRISPR-Cas only shows a *cas6f* (SHEWT2_03436) and a *csy*3 (SHEWT2_03436) genes with low evidence for a CRISPR cluster according to the CRISPR-Cas++ viewer. When searching the reported spacers, in the spacer database of the CRISPRCas++ database, most of the repeats do not show similarity to virus sequences or they are similar to plasmid sequences from *S*. *putrefaciens* strains. To the best of our knowledge, viral taxa within the hard-rock subsurface have not been described so far. Viruses have shown to be present in extreme environments [67], thus viruses in the subsurface could also be thriving on these microbial communities as the presence of CRISPR signatures suggest. 

A putative plasmid was predicted (GCA_902726625.2) with 20 genes, where 4 of them are hypothetical proteins [14]. Annotated genes include four transposases, two ribosomal proteins and two DNA-directed RNA polymerase beta subunit. When searching the hypothetical proteins against the BLAST database only two (EEHMALJP_00017 and EEHMALJP_00018) returned hits against de NCBI database. EEHMALJP_00017 is similar to a fimbria-like protein, while EEHMALJP_00018 shows a Mob_pre (pfam1706) domain, which is involved in plasmid recombination [68]. In this case, most of the annotated sequences corresponded to ribosomal proteins, transposases or an RNA polymerase. It remains to be seen what role this extrachromosomal element could have in the subsurface.

#### 3.1.2. Gene Redundancy and Analysis of the Cobalamin Receptors

Several genes from the chromosome show a remarkably high number of copies (Supplementary Table S7) with 13 copies of the *btuB* (SHEWT2_00390, SHEWT2_01006, SHEWT2_01171, SHEWT2_01470, SHEWT2_01531, SHEWT2_01708, SHEWT2_01825, SHEWT2_01900, SHEWT2_02367, SHEWT2_02382, SHEWT2_02956 and SHEWT2_04015) gene, which translates into a receptor for extracellular vitamin B12. Other proteins of the vitamin B_12_ receptor transporter show redundancy such as *btu*D with six copies (SHEWT2_00535, SHEWT2_00798, SHEWT2_01078, SHEWT2_02296, SHEWT2_02436 and SHEWT2_02919) and *btu*F with two copies (SHEWT2_00223, SHEWT2_02656) genes, respectively. Vitamin B_12_ plays a significant role in several essential metabolic processes (i.e., DNA synthesis, fatty acid and/or amino acid metabolism). Not all microorganisms are able to synthesize it de novo and indeed *Shewanella* sp. T2.3D-1.1 does not possess the necessary genes for its synthesis. The synthesis pathway of vitamin B_12_ is very complex and has long been energetically demanding. Nevertheless, it has the genes for the so-called salvage pathway, which involves the uptake from the extracellular medium of intermediate forms of the vitamin and its synthesis from those [31]. The BtuBCDF transport system responsible for its capture shows a high number of copies in the chromosome of the strain T2.3D-1.1, except for *btu*C, which has not been annotated as so in the genome. When using the BtuC sequence from *S. oneidensis* MR-1 (WP_011071293.1) to search against the translated coding sequences of the strain T2.3D-1.1, a HmuU protein (CAD6363917.1) showed 81.32% of identity with a 99% of coverage. Therefore, it is possible that this gene could code for the missing protein. *Shewanella* sp. T2.3D-1.1’s *btuB* genes, generally show the same protein domains, such as TonB dependent protein, and iron transport-related domains such as FecA and CirA. TonB is a regulator for many receptors involved in iron uptake such as FecA and CirA, nickel uptake or vitamin B_12_, such as BtuB, among others [69]. Motif location can also give information about the similarities with other reference proteins. Using the MEME suite and the BtuB protein sequences of *E. coli* (CAD6020855.1) and *Shigella dysenteriae* (RIH46231.1) as references, similar motif structures could be observed in most of the copies (Supplementary Figure S1). To further analyse these sequences an alignment with BtuB proteins from other *Shewanella* representatives, *E. coli*’s and *S. dysenteriae*’s BtuB proteins was made, and a tree was inferred (Supplementary Figure S2). Although none of the *Shewanella* sp. T2.3D-1.1 copies are closely allocated to the reference one, domains and motifs seem to imply that most of these proteins are BtuB-like. Numerous paralogs of the BtuB transporter could provide competitive advantages regarding the different forms of the B_12_ vitamin as seen in *Bacteroides thetaiotaomicron* [70]; perhaps this could be the case for *Shewanella* sp. T2.3D-1.1

#### 3.1.3. Phylogenomic Analyses

As reported by the rRNA, 16S based taxonomy placed this isolate within the *Shewanella* genus showing a 99.43% similarity with the closest relative, *S. hafniensis* P010(T) [14]. To obtain a better taxonomic resolution of the isolate, several phylogenomic analyses were performed. The genomes of all the type strains of the *Shewanella* genus and *S. putrefaciens* CN-32 (Supplementary Table S1) were compared against the *Shewanella* sp. T2.3D-1.1 to obtain their ANI values. *S. putrefaciens* CN-32 was included because it was isolated from the subsurface [71], therefore being of great interest for our study. According to the ≥95% threshold [23] with ANI, the *S. putrefaciens* species would fall over the intraspecies threshold with a 99.25% (Supplementary Table S8). The dDDH values concur with the ANI results, since the only genomes that show higher values than the 70% interspecies threshold are the *S. putrefaciens*. Altogether, the T2.3D-1.1 strain seems to belong to the *S. putrefaciens* species (Supplementary Table S9). Additionally, the phylogenomic tree was also built using the genomes from *Shewanella* type strain and the genomes of all available *S. putrefaciens* strains from the NCBI database. A large portion of the *S. putrefaciens* species are clustered together including the type strains *S. putrefaciens* JCM 20190a and NBRC 3908, and our strain *Shewanella* sp. T2.3D-1.1, which reinforces the assignment of the strain T2.3D-1.1 as a member of the species *S. putrefaciens* (Supplementary Figure S3). 

**Figure 2:**
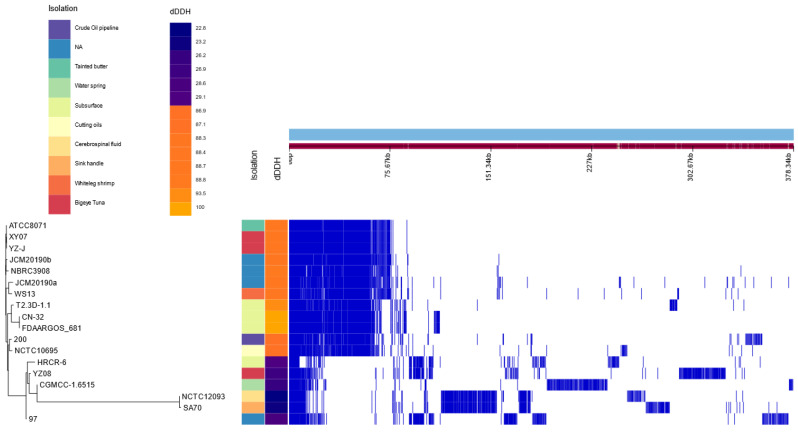
Graphical representation of the pangenome obtained from all available *S. putrefaciens* strains including *Shewanella* T2.3D-1.1. Additional columns show the isolation source and dDDH values when using *Shewanella* T2.3D-1.1 as the query genome. Isolation sources are represented in different colours. dDDH range from blue for the lowest values, to orange for the highest values using *Shewanella* sp. T2.3D-1.1 as the reference. Legend for each additional column is displayed on the top left corner.

A pangenome refers to the complete set of genes contained in all species of a clade. Only the genomes from the *S. putrefaciens* species, including *Shewanella* sp. T2.3D-1.1, were used to create a pangenome (Supplementary Table S1). There are considerable differences regarding the genomic content in these species. Out of the total of 18,915 genes, 397 can be considered as core genes, 0 genes classified as soft-core genes, 5023 shell genes and the remaining as cloud genes. Strains FDAARGOS-681, CN-32, HRCR-6 and T2.3D-1.1 have been isolated from subsurface environments. Particularly FDAARGOS-681, CN-32 and T2.3D-1.1 have 3609 genes in common (Figure 2) and higher dDDH values compared to the rest. Figure 2 shows two different clusters, one that includes the type strain *S. putrefaciens* JCM 20190^a^, and another one with the *S. putrefaciens* 97, SA70 and HRCR-6 strains among others. Thorell et al. (2019) have pointed out that many of the strains included in this second cluster have an incorrect species assignment according to their dDDH values [72]. Our results also show an incorrect species assignation for some strains of the *Shewanella* genus. Nevertheless, this further reinforces our designation of *Shewanella* sp. T2.3D-1.1 as a member of the *S. putrefaciens* species, since it is included inside the group where the type strain is assigned. 

It is remarkable how despite geographical distance between subsurface sampling points, there are microorganisms that share such a high genomic similarity. *S. putrefaciens* CN-32 was isolated at 250 m depth from shale-sandstone sequence in the Morrison formation in New Mexico [71]. *S. putrefaciens* FDAARGOS-681 was also isolated from subsurface rock at Cerro Negro in New Mexico [73]. *Shewanella* sp. T2.3D-1.1 was isolated in the Iberian Peninsula at a depth of 122 m in the subsurface of the IPB with a mineralogical composition dominated by quartz and illite [14,60]. 

Altogether, results from ANI and dDDH values coupled with the pangenome and the phylogenomic tree seem to imply that *Shewanella* T2.3D-1.1 belongs as a member of the *S. putrefaciens* species. Nevertheless, additional interesting results have arisen from our studies regarding the taxonomy of the *S. putrefaciens* species. Genomes assigned to the strains 97, CGMCC-1.6515, HRCR-6, NCTC12093, SA70, and YZ08 have displayed dDDH values that fall well below the intraspecies threshold (Supplementary Table S2). The phylogenomic tree built with all the *Shewanella* genomes mentioned in this article also shows that these strains do not allocate with the *S. putrefaciens* species cluster but instead can be found with *S. hafniensis* ATCC BAA-1207, *S. bicestrii* JAB-1 or clustered by themselves (Supplementary Figure S3). According to Thorell et al. (2019) strains SA70 and NCTC12093 belong to the *S. bicestrii* species and strains 97 and HRCR-6 have a wrong species assignation although no specific species assignation is mentioned by these authors [72]. Strains CGMCC-1.6515 and YZ08 have not been addressed in Thorell et al. (2019), but our results also suggest an incorrect taxonomic assignation. 

#### 3.1.4. Variant Calling Analysis between *Shewanella* sp. T2.3D-1.1 and *S. putrefaciens* CN-32

Once the closest genome from the sample was determined, differences between *Shewanella* sp. T2.3D-1.1 and *S. putrefaciens* CN-32 were studied through variant calling analysis. The alignment between both genomes reported 20,688 variants across the whole *S. putrefaciens* CN-32 chromosome, where 19,992 are classified as single nucleotide polymorphisms (SNPs), 357 are insertions and 319 are deletions. SNPs have a higher number of transitions over transversions. In the case of insertions and deletions, the mean length was 2.3 pb with a 5.3 of standard deviation. Every variant can have one or more predicted effects, and, in this case, the detected variants have 207,503 predicted effects. These effects can be classified according to their type, region, impact or functional class. Most of the effects are located on the upstream or downstream regions, have a modifier effect and their functional class is silent (Table 1). Finally, variants were annotated into specific genes and classified into metabolic routes according to the KEGG database. As a result, 3400 genes were obtained, of which only 1013 were classified in KEGG pathways. A total of 539 genes were clustered inside the overview pathway known as “metabolic pathways”. Other KEGG routes, such as “biosynthesis of secondary metabolites” and “microbial metabolism in diverse environments, hold a big part of these genes with 241 and 137, respectively (Supplementary Tables S5 and S10). 

Pathways of relevance to the biogeochemical cycles, such as sulphur metabolism, nitrogen metabolism or flavin synthesis, have genes with variants. In the nitrogen metabolism, 14 different genes involved in it show variants with its predicted effects (Supplementary Tables S7 and S8). In the case of the sulphur metabolism, only one gene shows a high impact effect, which is one of the copies of the *cys*Q gene. These two effects with high impact are classified as frameshift variants and could render this protein unfunctional. The genes involved in the EET were not included in any KEGG pathway except for the genes necessary for the synthesis of flavins which do not show any variants with high impact effects (Supplementary Tables S7 and S8). Genes involved in the EET sensu stricto do show variants with high impact effects. Two copies out of the four cytochromes c (*omc*A/*mtr*C) genes show variants with high impact effects, predominantly located in the upstream region. The NILIPAHB_00866 (*omc*A/*mtr*C) gene has three effects classified as high impact, where two of them show a frameshift variant effect and the other one is related to the gain of a stop codon. While the other gene NILIPAHB_00770 (*omc*A/*mtr*C) has four effects with high impact and all of them are marked as frameshift variants. In addition, the *ush*A (SHEWT2_01561) gene has variants with two high impact effects with a frameshift prediction (Supplementary Table S7). This gene codes a protein that hydrolyzes FAD to FMN, which is the predominant flavin in the extracellular medium for *Shewanella*. When the UshA protein is defective, the FAD takes the place of FMN as the predominant flavin with very little effect on the viability of the cell [74,75]. These three genes could possibly lose their functionality as a result of these variants, although in vitro experiments will be necessary to test whether these effects have consequences or not. 

### 3.2. Phenotypic Characterization

#### 3.2.1. Metal Tolerance and Electron Acceptors

The subsurface of the IPB is well known as a reservoir of massive metallic sulphides, being one of the largest on Earth [56]. Due to the high metal content of Río Tinto, it was deemed necessary to study potential mechanisms in *Shewanella* sp. T2.3D-1.1 [3]. *Shewanella* sp. T2.3D-1.1 was able to grow in the presence of 7.5 mM of Pb^2+^, 5 mM of Cr^3+^ and Cu^2+^ and 1 mM of Cd^2+,^ Co^2+,^ Ni^2+^ and Zn^2+^. Other *S. putrefaciens* strains showed higher tolerance for metals such as Cd^2+^ (27.2 mM), Zn^2+^ (18.6 mM), Co^2+^ (38.5 mM) and Cu^2+^ (31.4 mM) [76]. Since *Shewanella* sp. T2.3D-1.1 was isolated from hard-rock samples, the constitutive heavy metal tolerance might be lower than isolates facing higher concentrations of metals in solution. Similar to *Shewanella* sp. T2.3D-1.1, *S*. *putrefaciens* CN-32, shows tolerance values for toxic metals such as Cr^6+^ of 1 mM [77]. As mentioned, both strains were isolated from the deep subsurface, which may explain why their heavy metal tolerance is lower than other isolates from aquatic environments. Additional genes for metal resistance were searched and identified in the chromosome of *Shewanella* sp. T2.3D-1.1 [14] (Supplementary Table S9). These genes include arsenate or mercury reductases such as *ars*C (SHEWT2_01314 and SHEWT2_01319), *mer*A (SHEWT2_03691) and pumps such as *ars*B (SHEWT2_01318) and *czc*ABC (SHEWT2_01068, SHEWT2_02213, SHEWT2_03138, SHEWT2_03846, SHEWT2_04034, SHEWT2_04053, SHEWT2_04061, SHEWT2_04033 and SHEWT2_03848). The genus *Shewanella* is characterized by its environmental versatility due to its wide range of electron acceptors [16]. As mentioned, we found relevant genes for the reduction of a wide diversity electron acceptors in the chromosome of *Shewanella* sp. T2.3D-1.1 [14]. Accordingly, *Shewanella* sp. T2.3D-1.1 has grown in cultures with DMSO, Trimethylamine N-oxide (TMAO), NO_3_^−^ and fumarate, S_2_O_3_^−^ and S_4_O_6_^2−^ as electron acceptors. When *Shewanella* sp. T2.3D-1.1 was grown with fumarate (10 mM) as the electron acceptor, with either pyruvate (20 mM) or lactate (20 mM) as electron donors, H_2_ was detected in the headspace of the serum bottle after 37 days (Figure 1). With pyruvate, the partial pressure of H_2_ in 100 μL in the headspace was 17%, while for lactate it was 44%. Since lactate is more reduced than pyruvate, a higher partial pressure of H_2_ was to be expected. 

The reduction of DMSO and TMAO produces Trimethyl amine (TMA) and DMS, respectively, volatile molecules common in aquatic environments where the *Shewanella* genus has been studied more often. TMAO has been described to increase the production of flavins in *Shewanella* enhancing the EET [78]. Similarly, DMSO has also been reported to increase flavin excretion in *S. oneidensis* MR-1 therefore promoting the EET as well as the reduction of solid electron acceptors [79]. Electron acceptors such as DMSO or TMAO have not been addressed, to the best of our knowledge, in the deep subsurface context. Hence, their utilization by *Shewanella* sp. T2.3D-1.1 and role in the subsurface environment remains unclear. 

NO_3_^−^ is a widely spread electron acceptor and a common phenotypic trait for prokaryotes. It has been detected in the IPB’s subsurface [60] and acidic mine pit lakes [80]. Although the source of this ion is unknown, other studies have reported the genetic potential to reduce NO_3_^−^ to NO_2_^−^ and NO_2_^−^ to NH_4_^+^ in the continental subsurface [5]. Similarly, *Shewanella* sp. T2.3D-1.1 possesses the necessary genes to produce NH_4_^+^, which can then be used as a source of energy and/or nitrogen for other microorganisms.

As mentioned, in the genome of *Shewanella* sp. T2.3D-1.1, we found genes encoding proteins for the reduction of S^0^, S_2_O_3_^2−^ and S_6_O_4_^2−^ to H_2_S (Figure 1). H_2_S can be used by other bacteria as an electron donor or when reacting with FeS, promoting the production of H_2_ and precipitation of FeS_2_ [81]. H_2_ production has also been described through fermentation in *S. oneidensis* MR-1 [42], which shows a very similar composition regarding fermentation genes compared to *Shewanella* sp. T2.3D-1.1. Therefore, it is possible that *Shewanella* sp. T2.3D-1.1 could be producing H_2_ in the subsurface through fermentation. The fermentation of lactate or pyruvate by *Shewanella* produces acetate and formate, which is then further oxidized to H^+^ and CO_2_ [82,83]. In fact, both formate and acetate have been detected in the subsurface of the IPB [60]. An H_2_-producing microorganism could benefit microbial communities in the subsurface promoting growth despite the extreme conditions [84]. Biological production of H_2_ in the subsurface of the IPB has also been detected [60,84]. Since microorganisms in the subsurface do not have access to sunlight photosynthesis, it is not a feasible source of energy and carbon. Therefore, microorganisms would depend on other sources of energy [85], such as H_2_. H_2_ can be used as an electron donor for many microorganisms present in the IPB’s subsurface such methanogenic archaea and acetogenic bacteria [84]. 

The physiological data confirms what the annotated genes predicted, *Shewanella* sp. T2.3D-1.1 can use a wide variety of electron acceptors and produce H_2_ (Figure 1), a possible source of energy for the rest of microorganisms of the deep subsurface. 

#### 3.2.2. Iron Oxidation and Reduction Assays

**Figure 3:**
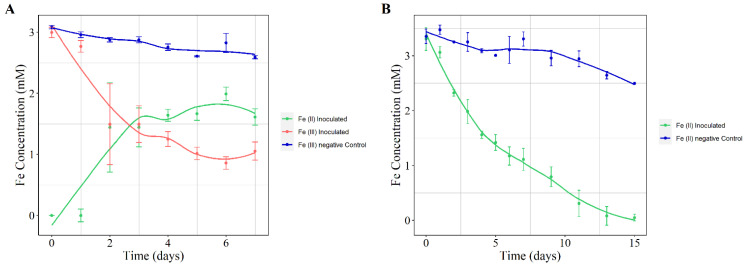
Reduction (**A**) and oxidation (**B**) of Fe of *Shewanella* sp. T2.3D-1.1. (**A**) In blue, concentration of Fe^3+^ in the control bottle; in red, evolution of Fe^3+^ in the cultures; in green, concentration of reduced Fe^2+^ in the inoculated cultures. (**B**) In blue, concentration of Fe^2+^ in the control (absence of microorganism); in green, concentration of Fe^2+^ in the inoculated cultures.

Reduction of Fe^3+^ is a well-known feature among the *Shewanella* genus [36,86], including both soluble and non-soluble forms. To test this activity, *Shewanella* sp. T2.3D-1.1 was exposed to Fe^3+^-citrate as an electron acceptor to evaluate its Fe reducing capabilities. The initial measured concentration of Fe^3+^ was 2.99 mM. Reduction of Fe^3+^-citrate was clearly detected after 48 h with 1.44 mM of Fe^2+^ detected in solution. On the seventh day, a 65% of the added Fe^3+^ was reduced to Fe^2+^ by *Shewanella* sp. T2.3D-1.1 (Figure 3A). Throughout the experiment, white precipitates were observed on the bottom of the inoculated bottles but not on the controls. These precipitates could be the product of Fe^2+^ reacting to the phosphate or carbonate of the medium, producing vivianite and/or siderite respectively [38]. Vivianite has several applications ranging from heavy metal immobilization, dichlorination of CCl_4_, phosphorus sequestration or as a slow-release fertilizer [87]. Further studies should determine the nature of these precipitates and its possible biotechnological applications.

Several authors have studied the interaction between NO_3_^−^ reduction and Fe redox reactions both in mineral form and in coating the membrane of *Shewanella* spp. [88,89]. These studies seem to imply that the *Shewanella* genus could in principle perform the abiotic Nitrate Dependent Fe Oxidation (NDFO). The genome of *Shewanella* sp. T2.3D-1.1 does not contain any known genes related to the direct oxidation of Fe^2+^. However, since it can reduce NO_3_^−^, *Shewanella* sp. T2.3D-1.1 could perform the NDFO. To resolve this, *Shewanella* sp. T2.3D-1.1 was grown under NDFO conditions, with lactate as electron donor NO_3_^−^ as electron acceptor with soluble Fe^2+^ in the medium. In the NDFO, NO_3_^−^ is reduced to NO_2_^−^ which then oxidizes Fe^2+^ to Fe^3+^ (Figure 1). In the case of iron oxidation, due to the possible precipitation of vivianite and siderite, the initial concentration of Fe^2+^ was 3.3 mM. In these conditions, *Shewanella* sp. T2.3D-1.1 was able to completely oxidize all the Fe^2+^ in 13 days (Figure 3B).

This is the first time that a member of the genus *Shewanella* has been described to be able to perform the NDFO (besides the utilization of soluble forms of Fe^2+^). It is still unknown whether microorganisms capable of doing the NDFO in heterotrophic conditions carry out an enzymatic oxidation of the Fe^2+^ (mixotrophs) or the Fe^2+^ reacts abiotically with the nitrite and nitric oxide (chemodenitrifiers) [90,91]. Either way, this is a process of great interest in the context of the IPB, since it has been hypothesized that in the absence of O_2_, it could be responsible for the oxidation of FeS_2_ in the subterranean bioreactor, creating the extreme conditions detected in Río Tinto [18,40]. 

*Shewanella* has been reported to reduce Fe minerals as well as Fe soluble forms [36,92]. Both the reduction and oxidation of Fe could act as different paths to dissolve minerals and further increase the content of soluble metals in the underwater reservoir. Fe^3+^ can react with the S^2−^ of the pyrite (FeS_2_) oxidizing it to SO_4_^2−^ while Fe^3+^ is reduced to Fe^2+^ and protons are released as well [93,94]. The Fe^2+^ would be oxidized indirectly through the NDFO by *Shewanella* sp. T2.3D-1.1 to Fe^3+^ which would dissolve more pyrite, releasing even more Fe^2+^ into solution [40] creating a feedback loop. This reaction also releases protons which allows Fe^3+^ to stay in solution, increasing its buffer effect and keeping a low pH. The Fe^3+^ buffer keeps the pH of the water low allowing metals such as iron itself to remain soluble [2]. In the IPBSL drilling project, NO_3_^−^ was detected throughout the entire BH10 borehole as well as sugars and compounds that could act as possible carbon sources [60]. Therefore, the conditions in the subsurface of the IPB would enable the NDFO, resulting in the aforementioned loop [40].

## 4. Conclusions 

According to the performed phylogenomic analysis, we propose that the strain *Shewanella* sp. T2.3D-1.1 should be named as *Shewanella putrefaciens* T2.3D-1.1. The strain isolated from the IPB and described in this study is remarkably close to other strains from the same species that also have been isolated from the subsurface. *S. putrefaciens* T2.3D-1.1’s genome has a high number of putative B_12_ transporters, possibly denoting the requirement of other microorganisms to produce it. Its possible role within biogeochemical cycles of sulphur and nitrogen has been narrowed as a dissimilatory reductor of NO_3_^-^, NH_2_OH, DMSO and H_2_ and H_2_S producer. More importantly, genes involved in the EET have been detected as well, meaning that this mechanism could be performed by *S. putrefaciens* T2.3D-1.1. This strain can reduce iron and oxidize it through the NDFO, participating actively in the subsurface iron cycle (Figure 1). 

The deep subsurface is no longer dismissed as devoid of life, in fact, bacteria and archaea are thought to have most of their biomass on such environments [95]. The IPB is a deep subsurface environment which has been thoroughly characterized and studied, allowing to understand how microorganisms can both survive and transform their surroundings [6,19]. *S. putrefaciens* T2.3D-1.1 could play a key role in the operation of the carbon, nitrogen, sulphur, and iron cycles, sustaining microbial populations and modifying the mineral content of the subsurface with its versatile metabolic activities.

## Figures and Tables

**Table 1:** Summary of detected variants between *S. putrefaciens* CN-32 and *Shewanella* sp. T2.3D-1.1.

Type				Region	
Type	Count	Percent	Type	Count	Percent
Conservative inframe deletion	8	0.00%	Downstream	93.502	45.16%
Conservative inframe insertion	19	0.01%	Exon	17.776	8.59%
Disruptive inframe deletion	24	0.01%	Intergenic	2.893	1.40%
Disruptive inframe insertion	25	0.01%	Splice site region	10	0.01%
Downstream gene variant	93.502	45.15%	Upstream	92.872	44.85%
Upstream gene variant	92.872	44.85%	**Number of effects by impact**		
Intergenic region	2.893	1.40%	**Type**	**Count**	**Percent**
Missense variant	3.311	1.60%	High	254	0.12%
Noncoding transcript exon variant	12	0.01%	Low	14.134	6.83%
Splice region variant	16	0.01%	Moderate	3.386	1.64%
Start lost	5	0.00%	Modifier	189.279	91.41%
Stop gained	26	0.01%	**Number of effects by functional class**		
Stop lost	4	0.00%	**Type**	**Count**	**Percent**
Stop retained variant	10	0.01%	Missense	3.318	18.99%
Synonymous variant	14.124	6.82%	Nonsense	24	0.14%
Frameshift variant	222	0.11%	Silent	14.134	80.88%

## Data Availability

The data presented in this study are available in the article and supplementary material.

## Supplementary Materials

The following supporting information can be downloaded at: https://www.mdpi.com/article/10.3390/microorganisms10081585/s1, Supplementary Figure S1: Phylogenomic tree built with UBCG using all the *Shewanella* genomes available in this article; Supplementary Figure S2: Motifs present in the BtuB copies of *Shewanella* T2.3D-1.1 compared to reference proteins; Supplementary Figure S3:Phylogenetic tree of all BtuB proteins from the *Shewanella* genus; Supplementary Table S1: *Shewanella* genomes used throughout this article from the GenBank database; Supplementary Table S2: Metadata used for Figure 2; Supplementary Table S3: Output obtained from the variant calling analysis.; Supplementary Table S4: Variant calling analysis results classified and mapped to *Shewanella putrefaciens* CN-32; Supplementary Table S5: Genes obtained that showed variants were further classified into metabolic pathways; Supplementary Table S6: Genes found in the chromosome of *Shewanella* T2.3D-1.1 and that have been mentioned throughout this article; Supplementary Table S7: Genes that show 4 copies or more in *Shewanella* T2.3D-1.1’s genome; Supplementary Table S8: ANI values obtained from comparing *Shewanella* T2.3D-1.1 to type strain genomes of the *Shewanella* genus; Supplementary Table S9: dDDH values obtained from comparing *Shewanella* T2.3D-1.1 to *Shewanella* genus type strains with three different formulas; Supplementary Table S10: KEGG mapper information of each gene found in the variant calling analysis from *Shewanella putrefaciens* CN-32; Supplementary Material S1: CRISPR-Cas Meta tool output when using *Shewanella* T2.3D-1.1’s chromosome as input; Supplementary Material S2: Aminoacid sequences of all the BtuB proteins from *Shewanella* T2.3D-1.1 and reference proteins from *Escherichia coli* and *Shigella dysenteriae*.
