# Supplementary material for: Shewanella sp. T2.3D-1.1 a Novel Microorganism Sustaining the Iron Cycle in the Deep Subsurface of the Iberian Pyrite Belt
Source: Microorganisms. 2022 Aug 6;10(8):1585. doi: 10.3390/microorganisms10081585 (PMC9415397; doi:10.3390/microorganisms10081585)
Supplement: Supplementary file 1 [file microorganisms-10-01585-s001.zip › Supplement Figures.pdf]

Supplementary Materials:

Shewanella sp. T2.3D-1.1 a novel microorganism sustaining the Iron cycle in the deep subsurface of the Iberian Pyrite Belt

Guillermo Mateos [1,*], Adrián Martínez Bonilla [1], Sofía de Francisco de Polanco [2], José M. Martínez [1], Cristina Escudero [3], Nuria Rodríguez [1,3], Irene Sánchez-Andrea [4] and Ricardo Amils [1,3]

[1] Centro de Biología Molecular Severo Ochoa, Nicolás Cabrera 1, 28049 Madrid, Spain
[2] Centro de Investigaciones Biológicas, Ramiro de Maeztu 9, 28040 Madrid, Spain
[3] Centro de Astrobiología (CAB-INTA), 28850 Torrejón de Ardoz, Spain
[4] Laboratory of Microbiology, Wageningen University & Research, Stippeneng, 46708 Wageningen, The Netherlands
* Correspondence: gmateos@cbm.csic.es

Supplementary Figure S1. UBCG tree built with the full set of genomes of the article. GSI index is displayed below branches in black. Distance is displayed on top of each branch in green. *Shewanella* T2.3D-1.1 has been highlighted with bold font.

Supplementary Figure S2. MEME graphical results of the motifs present in the BtuB proteins from *Shewanella* T2.3D-1.1 compared to *Shigella dysenteriae* (RIH4623.1) and *Escherichia coli* (CAD6020855.1).

Supplementary Figure S3. Phylogenetic tree of all available BtuB *Shewanella* sequences in the GenBank database with CLUSTAL-OMEGA. It also includes reference proteins from *E. coli* and *S. dysenteriae*. Distances are displayed as number on every branch.

Supplementary Table S1. Genomes obtained from the GenBank database that have been used in the different analyses of this article. All the type species have been marked with a bold superscripted "**T**". **Species** column indicates the species of the genome, **Strain** column is for the strain name and the **Assembly Accesion** column is for de accession code for the GenBank database.

Supplementary Table S2. Metadata employed for the elaboration of the Pangenome figure for the. This information is displayed in **Figure 2**. The **Name** column contains the strain names for every single *Shewanella putrefaciens* strain employed. The **Isolation** column has the available data on the source of each microorganism, where NA stands for Not Available. The **dDDH** column has the data for the Digital DNA-DNA Hybridization comparing *Shewanella* sp. T2.3D-1.1 to every single genome annotated as *Shewanella putrefaciens* available in the GenBank database.

Supplementary Table S3. Raw results of the variant calling analysis using *Shewanella putrefaciens* CN-32 as the reference against *Shewanella* T2.3D-1.1.

Supplementary Table S4. Results from the variant calling analysis classified and mapped to the *Shewanella putrefaciens* CN-32 chromosome. Columns with the "variant_" prefix indicate the type of variant that affects a particular gene or sequence.

Supplementary Table S5. ClusterProfiler classification of genes according to the Kegg.db on R. Pathways that are missing information were obtained through the KEGG Mapper tool using the genome of *Shewanella* sp T2.3D-1.1 and comparing it to *Shewanella putrefaciens* CN-32.

Supplementary Table S6. Genes found in *Shewanella* T2.3D-1.1's genome that have been mentioned throughout the article. Different row colours are determined by the associated pathway from the last column.

Supplementary Table S7. Genes that have 4 or more copies in the chromosome of *Shewanella* T2.3D-1.1.

Supplementary Table S8. ANI values calculated with *Shewanella* sp. T2.3D-1.1 as the reference against type strain genomes from the *Shewanella* genus.

Supplementary Table S9. dDDH results according to the three different formulas available on the web server. Each colour indicates different formulas from the online server used in the calculation. *Shewanella* sp. T2.3D-1.1 was set as the reference genome against type strains from the *Shewanella* genus.

Supplementary Table S10. KEGG mapper information on each for every gene found from *S. putrefaciens* CN-32 with variants. It contains the KEGG metabolic pathway in which each gene is mapped and the identifier and the name of the gene with a hyperlink to the database for further information.

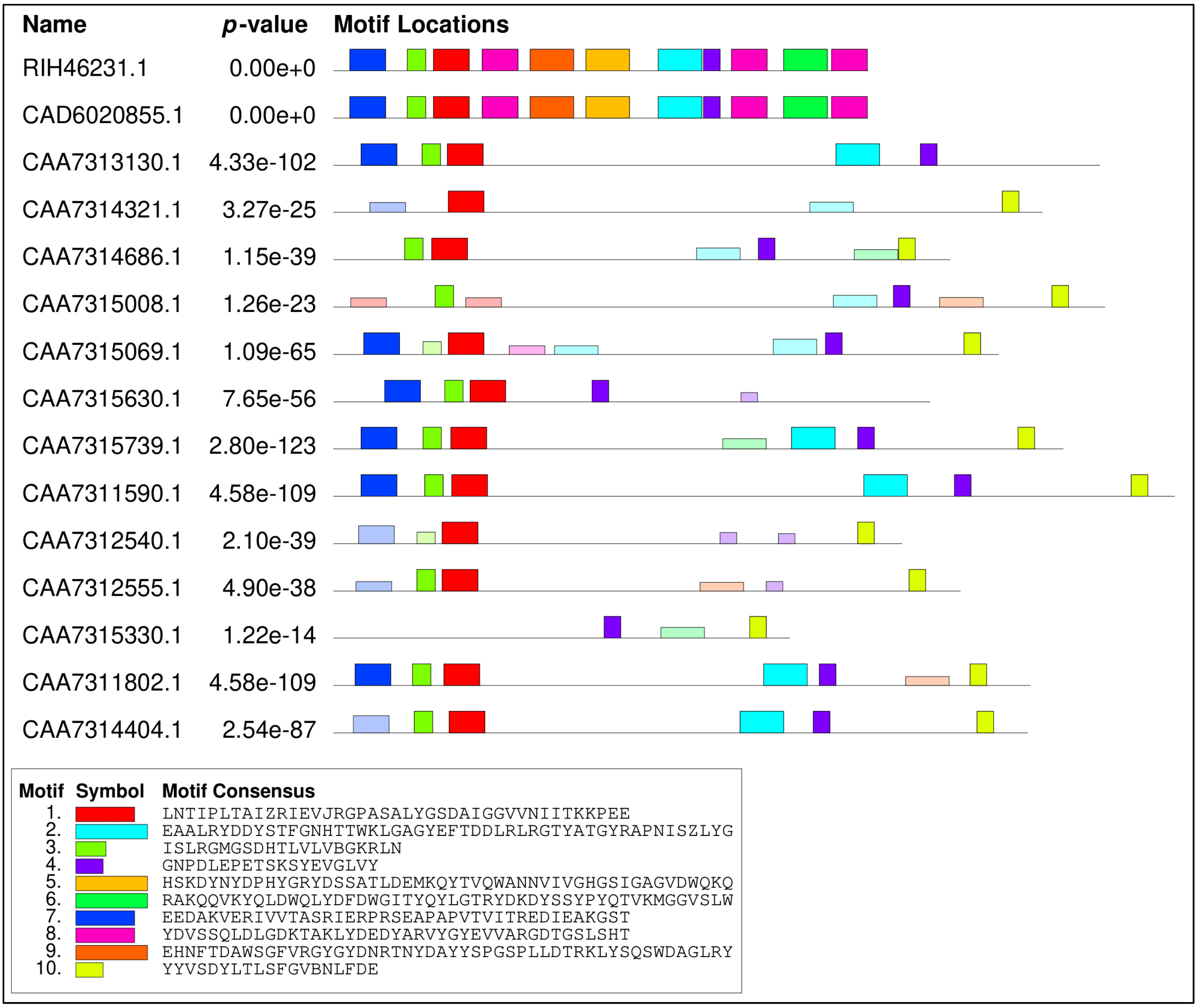

Supplementary Figure S1. MEME graphical results of the motifs present in the BtuB proteins from *Shewanella* T2.3D-1.1 compared to *S.dysenteriae* (RIH4623.1) and *E.coli* (CAD6020855.1).

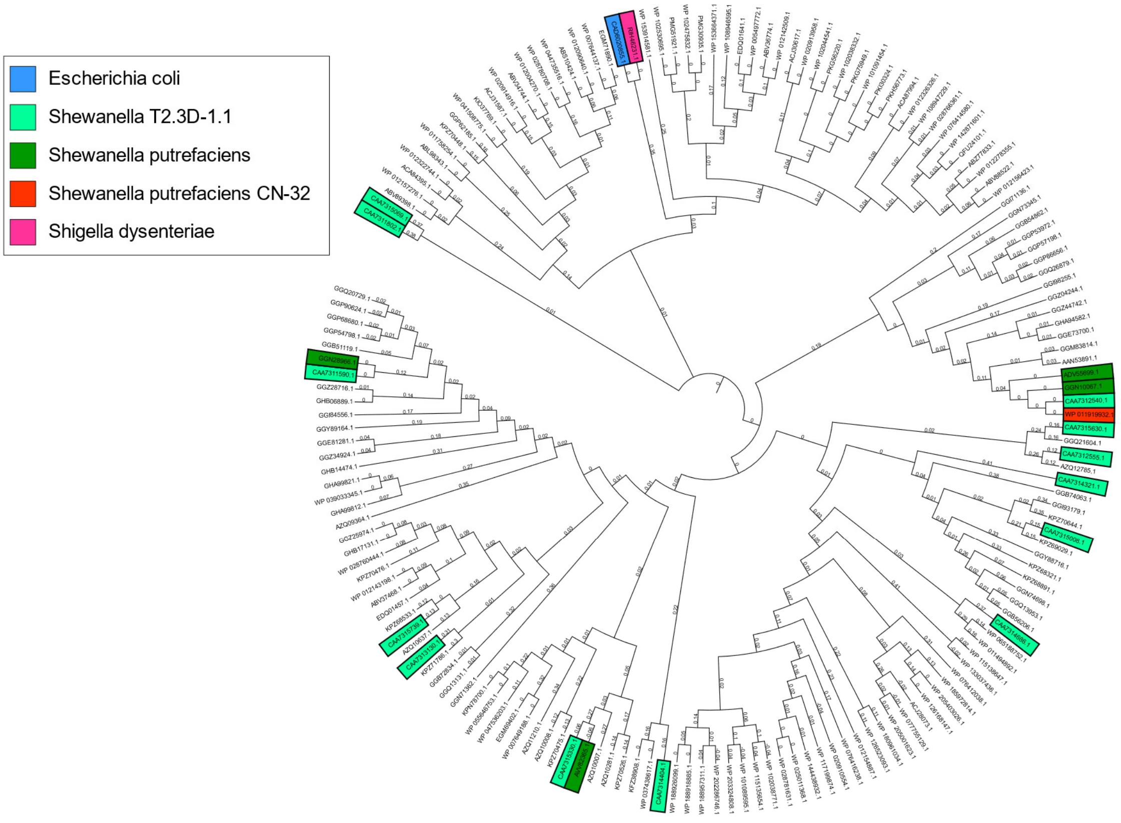

Supplementary figure S2. Phylogenetic tree of all available BtuB *Shewanella* sequences in the GenBank database with CLUSTAL-OMEGA. It also includes reference proteins from *E. coli* and *S. dysenteriae*. Distances are displayed as number on every branch.

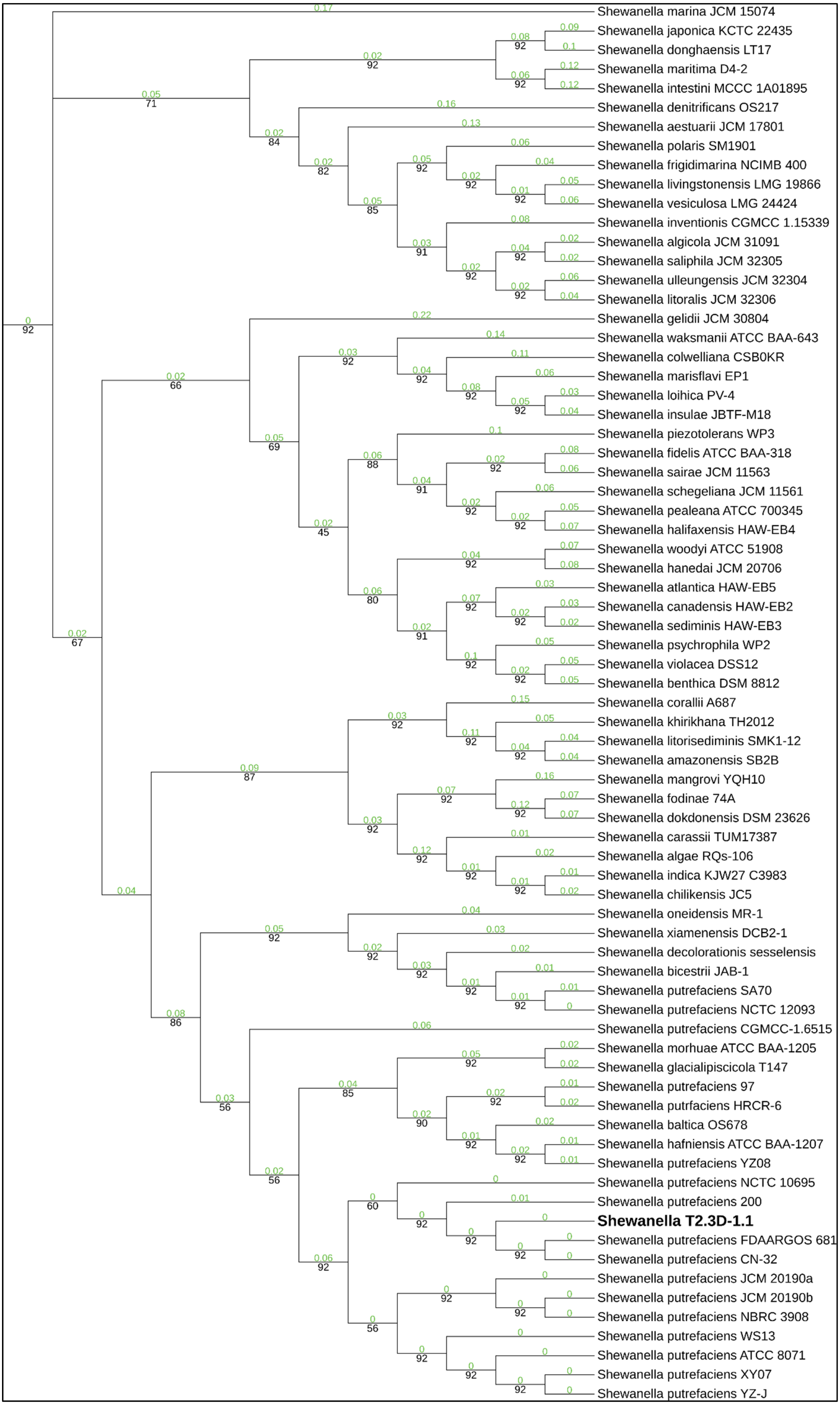
Shewanella marina JCM 15074
Shewanella japonica KCTC 22435
Shewanella donghaensis LT17
Shewanella maritima D4-2
Shewanella intestini MCCC 1A01895
Shewanella denitrificans OS217
Shewanella aestuarii JCM 17801
Shewanella polaris SM1901
Shewanella frigidimarina NCIMB 400
Shewanella livingstonensis LMG 19866
Shewanella vesiculosa LMG 24424
Shewanella inventionis CGMCC 1.15339
Shewanella algicola JCM 31091
Shewanella saliphila JCM 32305
Shewanella ulleungensis JCM 32304
Shewanella litoralis JCM 32306
Shewanella gelidii JCM 30804
Shewanella waksmanii ATCC BAA-643
Shewanella colwelliana CSB0KR
Shewanella marisflavi EP1
Shewanella loihica PV-4
Shewanella insulae JBTF-M18
Shewanella piezotolerans WP3
Shewanella fidelis ATCC BAA-318
Shewanella sairae JCM 11563
Shewanella schegeliana JCM 11561
Shewanella pealeana ATCC 700345
Shewanella halifaxensis HAW-EB4
Shewanella woodyi ATCC 51908
Shewanella hanedai JCM 20706
Shewanella atlantica HAW-EB5
Shewanella canadensis HAW-EB2
Shewanella sediminis HAW-EB3
Shewanella psychrophila WP2
Shewanella violacea DSS12
Shewanella benthica DSM 8812
Shewanella corallii A687
Shewanella khirikhana TH2012
Shewanella litorisediminis SMK1-12
Shewanella amazonensis SB2B
Shewanella mangrovi YQH10
Shewanella fodinae 74A
Shewanella dokdonensis DSM 23626
Shewanella carassii TUM17387
Shewanella algae RQs-106
Shewanella indica KJW27 C3983
Shewanella chilikensis JC5
Shewanella oneidensis MR-1
Shewanella xiamenensis DCB2-1
Shewanella decolorationis sesselensis
Shewanella bicestrii JAB-1
Shewanella putrefaciens SA70
Shewanella putrefaciens NCTC 12093
Shewanella putrefaciens CGMCC-1.6515
Shewanella morhuae ATCC BAA-1205
Shewanella glacialipiscicola T147
Shewanella putrefaciens 97
Shewanella putrfaciens HRCR-6
Shewanella baltica OS678
Shewanella hafniensis ATCC BAA-1207
Shewanella putrefaciens YZ08
Shewanella putrefaciens NCTC 10695
Shewanella putrefaciens 200
Shewanella T2.3D-1.1
Shewanella putrefaciens FDAARGOS 681
Shewanella putrefaciens CN-32
Shewanella putrefaciens JCM 20190a
Shewanella putrefaciens JCM 20190b
Shewanella putrefaciens NBRC 3908
Shewanella putrefaciens WS13
Shewanella putrefaciens ATCC 8071
Shewanella putrefaciens XY07
Shewanella putrefaciens YZ-J

Supplementary Figure S3. UBCG tree built with the full set of genomes of the article. GSI index is displayed below branches in black. Distance is displayed on top of each branch in green. *Shewanella* T2.3D-1.1 has been highlighted with bold font.
